# Predictors of burnout: the role of agency among obstetric providers in Kumasi, Ghana

**DOI:** 10.1080/16549716.2021.1978662

**Published:** 2021-09-29

**Authors:** Emma R. Lawrence, Michael Yeboah, Johnny Arthur-Komeh, Anna Stabnick, Sarah D. Rominski

**Affiliations:** a Department of Obstetrics & Gynecology, University of Michigan Medical School, Ann Arbor, Michigan, USA; b Directorate of Obstetrics and Gynaecology, Komfo Anokye Teaching Hospital, Kumasi, Ghana; c School of Public Health, University of Michigan, Ann Arbor, Michigan, USA

**Keywords:** Burnout, Ghana, obstetric provider, OBGYN, midwife, LMIC

## Abstract

Burnout rates among sub-Saharan African healthcare providers are high. In particular, obstetric providers experience unique stressors surrounding poor neonatal and maternal outcomes. This study explores predictors of burnout among obstetric providers at the Komfo Anokye Teaching Hospital (KATH) in Kumasi, Ghana. A survey was electronically distributed to midwives, house officers, and Obstetrician Gynecologists (OBGYNs) at KATH in Ghana. Demographic and clinical practice information was collected. Burnout was assessed using a 4-point Likert scale. To evaluate perceived agency caring for critically ill obstetric patients, participants responded to three statements and responses were summed to create an Agency Scale. Logistic regression was used to evaluate predictors of burnout. Marginal effects were calculated for factors significantly associated with burnout. Participants were 48 physicians and 222 midwives. Mean age was 32.4 years, mean years in practice was 6.5 years, and 83% had completed their medical training. Nearly half (49.6%) have personal experience with maternal mortality and 28.3% manage more than 5 maternal mortalities annually. The majority of participants (n = 152, 62%) reported feeling burned out from their work. After adjusting for role, number of annual maternal mortalities managed, and personal experience with maternal mortality, participants with more years in practice were 15.8% more likely to report being burned out (marginal effect = 0.158). Even after adjusting for years in practice, participants who scored higher on the Agency Scale had a significantly lower likelihood of reporting burnout (OR 0.76, 95% CI 0.66–0.88, p < 0.001). For each step up the Agency Scale, participants were 6.4% less likely to report they felt burned out. Rates of burnout are high among obstetric providers, particularly among providers who have practiced longer. Supporting provider agency to manage critically ill patients may reduce burnout rates.

## Background

Burnout is a syndrome of mental, physical and emotional exhaustion [1]. Healthcare workers are at a particularly high risk of burnout due to exposure to repeated emotional, physical, and psychological stressors [2]. In studies of healthcare providers in Sub-Saharan Africa, burnout rates are high – ranging from 40 to 80% – and are associated with negative work environments, professional conflicts, emotional distress, and low social support [3]. In particular, obstetric providers experience unique stressors surrounding poor neonatal and maternal outcomes. These stressors are compounded in low- and middle-income countries (LMICs) [4], where rates of maternal morbidity and mortality are highest and support for healthcare providers is inadequate. High levels of depersonalization, burnout, and emotional exhaustion are common [5,6].

Despite the importance of burnout among obstetric providers [5,6], little research has been done to identify and understand its predictors, especially in low-resource settings like Ghana. An overarching mixed methods study was performed among obstetric providers at the Komfo Anokye Teaching Hospital (KATH) in Kumasi, Ghana, focusing on the impact of managing frequent maternal mortalities [7]; semi-structured interviews demonstrated the importance of perceived control over clinical outcomes. Based on these findings, this study explores predictors of burnout among obstetric providers at KATH, with a specific focus on the role that agency plays in explaining rates of burnout. Better understanding of drivers of burnout among vulnerable obstetric providers, and the role of agency, can inform policies and protocols to better support them.

## Methods

A cross-sectional design was used to survey participants at KATH – a large tertiary care teaching hospital that performs approximately 10,000 deliveries each year with 100–150 maternal mortalities annually. Institutional Review Board approval was granted by KATH (AP/019/20) and the University of Michigan (HUM00175461). Participants were current obstetric providers at KATH – defined as midwives, OBGYNs, and house officers (new physicians who recently completed medical school) currently rotating on the OBGYN service.

An electronic survey was generated in REDCap and distributed electronically using KATH-specific WhatsApp groups, which are online groups used for communication. The members of the targeted WhatsApp groups included all midwives, OBGYNs, and house officers currently rotating on the OBGYN service at KATH. Electronically informed consent was obtained. Phone credit valued at $3.5 USD was provided as compensation.

Demographic information was collected, including age, gender, frequency of participation in religious activities, and personal experience with maternal mortality. Clinical practice information was collected, including trainee status, years in practice, and approximate deliveries (presented categorically as multiples of 25) and maternal mortalities (presented categorically as multiples of 5) managed annually. For analysis, years in clinical practice and annual maternal mortalities managed were dichotomized to greater >5 years versus ≤5 years. Measurement of burnout was adopted from prior studies in Nigeria [8] and South Africa [9]. Burnout was assessed using a 4-point Likert scale, ranging from strongly disagree (1) to strongly agree (4). Burnout responses were collapsed into two response categories for analysis: yes versus no. To evaluate perceived agency caring for critically ill obstetric patients, participants responded to three statements on a 4-point Likert scale ranging from strongly disagree (1) to strongly agree (4). An Agency Scale was created by summing the responses to those three statements, so the higher the score on the agency variable, the more the participant feels confident in their individual ability and their unit’s ability to handle maternal mortality.

Analysis was done in STATA. Bivariate analysis was used to evaluate predictors of burnout (yes vs no), using chi-squared with unadjusted odds ratios for categorical variables and t-tests and for continuous variables. Potential predictors evaluated by bivariate analysis included role, years practicing, age, gender, trainee status, number of annual maternal mortalities managed (dichotomized to a <5 vs 5 or more), personal experience with maternal mortality, scores on the Agency Scale. Variables significant in the bivariate model at p < 0.1 were included in a multivariate analysis. Predictors significant in the unadjusted bivariate analysis were role, years practicing, age, training status, number of annual maternal mortalities managed, and scores on the Agency Scale. Due to high collinearity between age, trainee status and years practicing, only years practicing was included in the final model. Despite being non-significant in the bivariate analysis, personal experience with maternal mortality was added to the final adjusted model due to author’s believing this is an important factor. Thus, the final adjusted logistic regression included role, years of practicing, number of annual maternal mortalities managed, personal experience with maternal mortality, and scores on the Agency Scale. For the final regression model, significance was defined as p < 0.05. Marginal effects were calculated for factors significantly associated with burnout.

## Results

Out of 390 potential participants who received the survey electronically, 270 participants completed the survey (response rate of 69%). Participants were 48 physicians and 222 midwives (Table 1). The total mean age was 32.4 years, mean years in practice was 6.5 years, and 82.5% had completed their medical training. Nearly half (49.6%) have personal experience with maternal mortality and 28.3% manage more than 5 maternal mortalities annually.

**Table 1.** Demographics

Characteristic	n (%) or Mean ± SD
Gender	
Male	43 (16.0)
Female	226 (84.0)
Age, years	32.4 ± 5.9
Marriage status	
Married	167 (62.3)
Not married	101 (37.7)
Children status	
Children	172 (63.9)
No children	97 (36.1)
Frequency of participation in religious activities	
One time per week or less	71 (29.2)
More than one time per week but less than daily	84 (34.6)
Daily	88 (36.2)
Medical training status	
Current trainee^a^	47 (17.5)
Completed training	222 (82.5)
Years in practice	6.5 ± 4.7
Approximate deliveries per year	
1–25	48 (17.8)
26–50	57 (21.2)
51–75	47 (17.5)
>75	117 (43.5)
Approximate maternal mortalities per year	
<5	185 (71.7)
5–10	48 (18.6)
11–16	10 (3.9)
>16	15 (5.8)
Personal experience (friend, family) with maternal death	
Yes	120 (49.6)
No	122 (50.4)

^a^house officer, resident, fellow, trainee midwife

**Figure 1.**
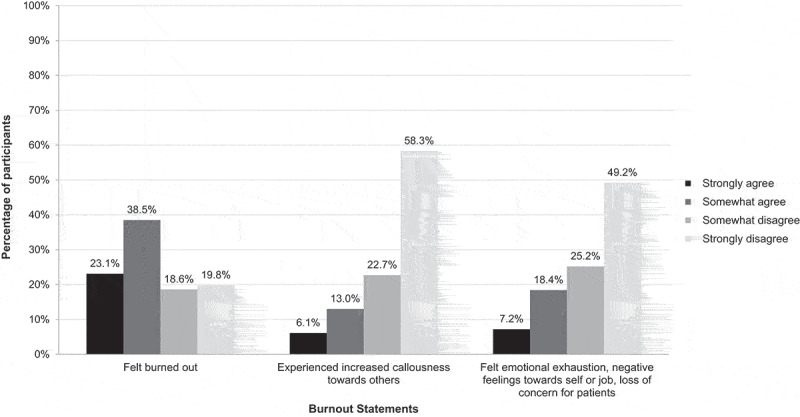
Burnout among obstetric providers

The majority of participants (n = 152, 61.6%) strongly agreed or somewhat agreed that they feel burned out from their work (Figure 1). When queried on other impacts of their obstetric practice, 19.1% (n = 47) have experienced increased callousness toward others and 25.6% (n = 64) have felt emotional exhaustion, negative feelings toward their self or job, or loss of concern for patients.

**Figure 2.**
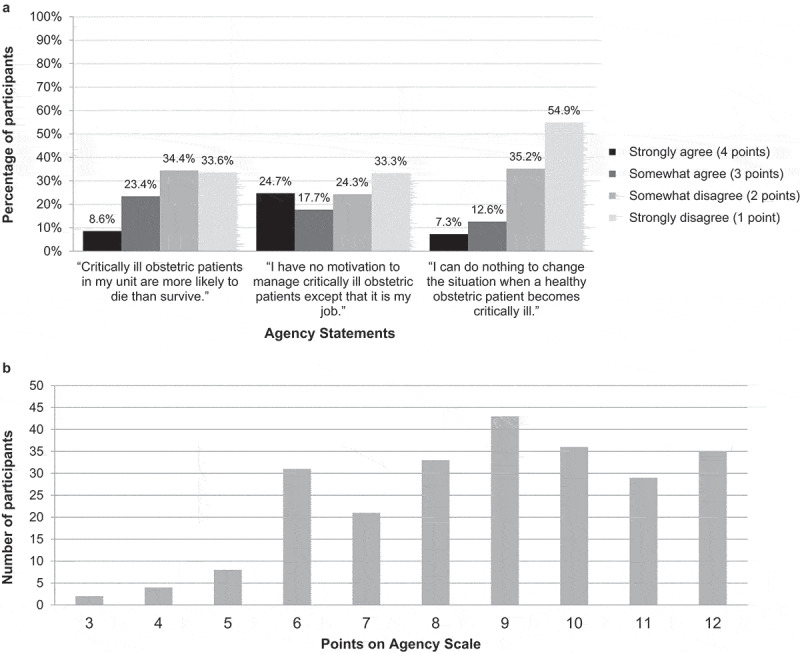
Attitudes toward caring for critically Ill obstetric patients

Regarding attitudes toward caring for critically ill obstetric patients, 32% (n = 78) agreed with the statement ‘critically ill obstetric patients in my unit are more likely to die than survive’; 42.4% (n = 103) agreed with the statement ‘I have no motivation to manage critically ill obstetric patients except that it is my job’; and 19.9% (n = 49) agreed with the statement ‘I can do nothing to change the situation when a healthy obstetric patient becomes critically ill’ (Figure 2A). As demonstrated in Figure 2B, responses to these questions were summed to create an Agency Scale, with possible scores ranging from 3 to 12. Scores on the Agency Scale ranged from 3 to 12 with a mean of 8.9 and standard deviation of 2.2.

In our final adjusted logistic regression (Table 2), participants with more years in practice were 15.8% more likely to report being burned out (marginal effect = 0.158). Even after adjusting for years in practice and all other variables in the model, participants who scored higher on the Agency Scale had a significantly lower likelihood of reporting burnout (adjusted OR 0.76, 95% CI 0.66–0.88, p < 0.001). For each step up the Agency Scale, participants were 6.4% less likely to report they felt burned out (marginal effects of Agency Scale = −.064).

**Table 2.** Predictors of burnout among obstetric providers, using adjusted logistic regression

Variable	Adjusted Odds Ratio	95% CI	Marginal Effect
Role			
Physician	0.5	0.2–1.0	
Midwife	REF		
Years in Clinical Practice			0.158
≤5 years	2.2	1.2–3.9^a^	
>5 years	REF		
Annual Maternal Mortalities Managed			
≤5	1.2	0.6–2.3	
>5	REF		
Personal Experience with Maternal Mortality			
No	1.2	0.7–2.2	
Yes	REF		
Agency Scale	0.7	0.6–0.8^b^	−0.064

^a^p<0.05

^b^p<0.001

## Discussion

Rates of burnout are high among obstetric providers in our study, with two-thirds agreeing that they feel burned out. Our study population is a high-risk group who practice in a tertiary care urban facility that is often overcrowded and under-resourced. Nearly half have personal experience with maternal mortality and one-quarter manage more than 5 maternal mortalities annually. Burnout rates in our study are comparable with findings in other physician and nurse populations in the literature [3,4,8,10]. Work-related stress is associated with mental health issues, including depression, anxiety, substance abuse, and suicidality [4]. Availability of counseling services, trainings on resilience and stress management, and opportunities for formal debriefings are rare for healthcare workers in LMICs [3]. Burnout contributes to emotional exhaustion, depression, decreased workplace satisfaction, and reduced quality of patient care [5,6].

We demonstrate that participants with more years in practice are more likely to report being burned out. This may be explained by cumulative stress and exposure to poor outcomes over time. In addition, more senior practitioners may be disproportionately called to manage complex patients with poor subsequent outcomes. They may also have additional responsibilities to balance at work, including supervision of junior practitioners, training of students, and departmental administrative roles. Low-income countries, including Ghana, deal with the unique issue of brain drain – where healthcare providers leave their country of origin to pursue improved living and working conditions in high-income countries [11]. Workplace stress and burnout are motivators of brain drain, which increases the strain on health systems and remaining providers [12,13].

In LMICs, practitioners may experience low agency to achieve good outcomes for patients due to overcrowding, understaffing, and lack of availability of resources needed to provide adequate patient care. In our study, participants who reported higher agency were less likely to report experiencing burnout. This suggests that interventions to support provider agency may decrease burnout rates in low-resource settings. There is little prior research on the relationship between agency and burnout, especially among healthcare providers [14].

Our study fills an important gap in the literature by exploring predictors of burnout among obstetric providers in a low-resource, high maternal mortality setting. This study was performed at a single urban tertiary hospital in Ghana, which may limit generalizability. However, the impact of our findings are supported by a high response rate and a large sample size of midwives, house officers, and OBGYNs at all levels of training. A full validated scale of burnout was not performed, which may lead to measurement error. Based on feedback from pretesting survey questions with a similar group of obstetrics providers at a different hospital, a shorter survey design was selected to maximize participation by busy practitioners. This approach and question selection was similarly utilized by studies on workplace burnout and depersonalization in Nigeria [8] and South Africa [9]. An anonymous electronic survey design was utilized to encourage honest responses.

## Conclusions

Burnout is an important issue to understand and address because it is detrimental to individuals and workplace environments, not only in Ghana, but also globally. We demonstrate that rates of burnout are significant among obstetric healthcare providers in a low-resource setting. Providers are more likely to feel burned out if they have more years in practice, and less likely to feel burned out if they have higher agency to care for patients. Globally, many hospitals in low-resource settings have high rates of poor maternal outcomes and high rates of burnout among obstetric providers. In these settings, building and supporting provider agency to manage critically ill patients may reduce burnout rates. Further research is needed to understand effective methods and policies to support provider agency in low-resource settings, which may include training to prepare providers to manage complex situations, debriefs, and workplace provider support when poor outcomes occur.

## Data Availability

The study dataset will be made available on email request to the corresponding author.

## Paper context

Rates of burnout are high among obstetric providers in Ghana, particularly among providers who have been in clinical practice longer. Obstetric providers with higher agency to care for critically ill obstetric patients had a significantly lower likelihood of reporting burnout. Globally, many hospitals in low-resource settings have high rates of poor maternal outcomes and high rates of burnout among obstetric providers. Thus, hospitals should enact policies to support provider agency to manage critically ill patients, including training, debriefs, and workplace support, which may reduce burnout rates.
